# Risk of End-Stage Renal Disease after Cancer Nephrectomy in Taiwan: A Nationwide Population-Based Study

**DOI:** 10.1371/journal.pone.0126965

**Published:** 2015-05-20

**Authors:** Wei-Yu Lin, Fu-Wen Liang, Tsung-Hsueh Lu

**Affiliations:** 1 Division of Urology, Department of Surgery, Chang Gung Memorial Hospital, Chiayi, Taiwan; 2 NCKU Research Center for Health Data and Department of Public Health, National Cheng Kung University, Tainan, Taiwan; Mario Negri Institute for Pharmacological Research and Azienda Ospedaliera Ospedali Riuniti di Bergamo, ITALY

## Abstract

**Background:**

The conclusions of population-based studies examining the risk of developing end-stage renal disease (ESRD) after nephrectomy among patients with renal cell carcinoma (RCC) remain inconclusive. In this study, we sought to examine whether patients with RCC undergoing radical nephrectomy (RN) have higher risk of ESRD compared to those undergoing partial nephrectomy (PN).

**Methods:**

Nationwide population-based retrospective cohort of 7670 patients with RCC who underwent RN or PN between 2000 and 2011 as recorded in the Taiwan National Health Insurance in-patient claims data were analyzed. The primary outcome of interest was the occurrence of ESRD requiring regular renal hemodialysis. Multivariable Cox proportional hazard regression model was performed to assess the risk.

**Findings:**

The median follow-up for the post-propensity matched cohort (1212 PN and 2424 RN) was 48 months. Seventy patients (2.9%) developed ESRD among those who underwent RN, for an incidence rate of 6.9 cases per 1000 person-years. In contrast, only 23 patients (1.9%) developed ESRD among patients who underwent PN, for an incidence rate of 5.5 cases per 1000 person-years. Despite the higher incidence rate of ESRD among RN, the aIRR (RN/PN) was 1.26 (95% CI 0.78-2.01), which was not statistically significant.

**Conclusions:**

This Taiwan nationwide population-based study suggests that patients with RCC undergoing RN do not have significantly higher risk of developing ESRD compared to those undergoing PN.

## Introduction

Surgical resection remains the gold standard of treatment for renal cell carcinoma (RCC). The guidelines suggested partial nephrectomy (PN) as the standard care for A1a RCC and as a viable option for T1b RCC [1,2]. Several hospital-based studies also indicated that patients undergoing radical nephrectomy (RN) are at a greater risk of chronic renal insufficiency than a similar cohort of patients undergoing PN [3–7]. A systematic review and meta-analysis of hospital-based studies indicate that PN for localized renal tumors provide a 61% risk reduction in chronic kidney disease compared to RN [8]. However, probably due to small number of participants observed, relatively few studies compared the effects of PN and RN on end-stage renal disease (ESRD).

Why is it important to emphasize the examination of ESRD as the primary endpoint? Well known for its association with increased risk of cardiovascular morbidity and mortality, ESRD is the ultimate adverse renal outcome [9]. Since patients with ESRD need renal replacement therapy, mainly regular hemodialysis, there is a substantial socio-economic impact on patients and their families. Thus, ESRD is a significant public health burden.

According to two recent reviews, despite increasing concerns on the prevention of ESRD, relatively few studies have examined the effects of nephrectomy on the occurrence of ESRD [10,11]. Only four population-based studies have specifically compared the risk of developing ESRD between PN and RN and the conclusions are not consistent [12–15]. Furthermore, no study on this issue has been done in Asian countries. We thus sought to use the Taiwan National Health Insurance (NHI) claims data to examine whether patients undergoing RN have higher risk of developing ESRD compared to PN patients among Chinese people.

## Methods

### Data source

The Taiwan NHI claims data for years 1996 through 2011 was used for analysis. The Taiwan NHI program was a mandatory, single-payer health insurance system in which all citizens are required to participate. The NHI program was implemented in 1995 and covered more than 99% of the 23 million citizens in Taiwan [16]. The NHI in-patient claims data were used to identify patients with RCC who received the first RN or PN. The in-patient claims data included encrypted ID, sex, date of birth, date of hospitalization, date of discharge, discharge diagnoses, procedures, characteristics of the hospital, and various kinds of payments.

### Study population

Patients with a primary discharge diagnosis of RCC (ICD-9-CM code 189) who underwent the first nephrectomy (ICD-9-CM code 55.4 for PN and 55.51 for RN) recorded in the in-patient claims data between 2000 and 2011 were included for analysis. Patients with CKD (ICD-9-CM code 585.3, 585.4, 585.5, and 585.9) and ESRD (ICD-9-CM code 585.6) before 2000 receiving the RN or PN were excluded.

### Main outcome

The Catastrophe Illness File of the NHI claims data was used to identify patients with ESRD who needed regular hemodialysis. To avoid severe financial difficulties for families coping with major injuries/illnesses, the NHI specified 31 categories of catastrophic illness (including cancers, hemophilia, systemic autoimmune diseases, and ESRD) exempted from co-payment. The attending physician of a patient diagnosed into the category of catastrophic illness under the NHI guidelines submitted related information in the application for catastrophic illness certificate. Applications were formally reviewed by a committee and once approved, the patients were exempted from co-payment [17].

### Covariates

Covariates included sex, age of patient with RCC receiving PN or RN, level and region of nephrectomy, co-morbidity (i.e., myocardial infarction, congestive heart failure, peripheral vascular disease, cerebrovascular disease, chronic pulmonary disease, ulcer disease, diabetes mellitus, and liver disease) and the Dartmouth-Manitoba's Charlson co-morbidity index (CCI).

### Statistical analysis

The propensity scores of patients undergoing PN was first estimated using a logistic regression model based on the patient and hospital characteristics. For any one patient receiving PN, two patient receiving RN were matched with the same propensity score to adjust the differences between patients undergoing PN and those undergoing RN.

The incidence rate (cases per 1000 person-years) of developing ESRD was then calculated among patients undergoing PN or RN. The adjusted incidence rate ratio (aIRR) and 95% confidence intervals (95% CI) between PN and RN was also calculated. Lastly, the Cox proportional hazard regression model was used to estimate the crude and adjusted hazard ratio (cHR and aHR, respectively) and 95% CI of ESRD occurrence.

## Results

A total of 7670 patients with RCC who underwent first nephrectomy (1212 PN and 6458 RN) were recorded in the NHI in-patient claims data between 2000 and 2011. There was a different distribution of geographical region and level of hospitals among patients who underwent PN and those who underwent RN (Table 1). Of 1212 patients who underwent PN, 86% were done in medical centers. However, among those who underwent RN, only 71% were performed in medical centers. After matching the propensity score, the characteristics of patients and hospitals between PN and RN were more compatible.

**Table 1:** Characteristics of patients with renal cell carcinoma who underwent partial (PN) or radical (RN) nephrectomy before and after propensity score matching in Taiwan, 2000–2011.

	Pre-propensity cohort			Post-propensity cohort		
Variables	PN	RN	*p* value	PN	RN	*p* value
**No**	1212	6458		1210	2420	
**Age, yr**			<.0001			0.477
** Mean (SD)**	57.3 (13.9)	60.1 (15.9)		57.3 (13.9)	56.9 (16.0)	
**Sex**			0.0005			0.761
** Male**	829 (68.4)	4080 (63.2)		827 (68.4)	1666 (68.8)	
** Female**	383 (31.6)	2378 (36.8)		383 (31.6)	754 (31.2)	
**Level of hospital**			<.0001			0.752
** Medical centers**	1045 (86.2)	4571 (70.8)		1043 (86.2)	2090 (86.4)	
** Regional hospitals**	160 (13.2)	1751 (27.1)		160 (13.2)	311 (12.8)	
** District hospital**	7 (0.6)	136 (2.1)		7 (0.6)	19 (0.8)	
**Region**			<.0001			0.880
** Taipei**	613 (50.6)	2714 (42.0)		611 (50.5)	1204 (49.8)	
** North**	135 (11.1)	884 (13.7)		135 (11.2)	260 (10.7)	
** Middle**	172 (14.2)	1105 (17.1)		172 (14.2)	337 (13.9)	
** Middle south**	114 (9.4)	686 (10.6)		114 (9.4)	250 (10.3)	
** Southern south**	172 (14.2)	986 (15.3)		172 (14.2)	361 (14.9)	
** East**	6 (0.5)	83 (1.3)		6 (0.5)	8 (0.3)	
**Co-morbidity**						
** CAD**	3 (0.3)	47 (0.7)	0.076	3 (0.3)	7 (0.3)	1.000
** CHF**	13 (1.1)	139 (2.2)	0.013	13 (1.1)	15 (0.6)	0.140
** PVD**	7 (0.6)	44 (0.7)	0.683	7 (0.6)	9 (0.4)	0.376
** CVD**	29 (2.4)	190 (2.9)	0.292	29 (2.4)	47 (1.9)	0.367
** CPD**	32 (2.6)	240 (3.7)	0.063	32 (2.6)	59 (2.4)	0.707
** UD**	71 (5.9)	352 (5.5)	0.569	69 (5.7)	110 (4.6)	0.129
** DM**	212 (17.5)	1003 (15.5)	0.086	210 (17.4)	414 (17.1)	0.852
** LD**	35 (2.9)	157 (2.4)	0.350	35 (2.9)	49 (2.0)	0.101
**CCI**			<.0001			<.0001
** 0**	0	0		0	0	
** 1**	0	0		0	0	
** 2**	846 (69.8)	4021 (62.3)		846 (69.9)	1532 (63.3)	
** ≥3**	366 (30.2)	2437 (37.7)		364 (30.1)	888 (36.7)	

Abbreviations: CAD, coronary arterial disease, CHF, congestive heart failure, PVD, peripheral vascular disease; CVD, cerebrovascular disease; CPD, chronic pulmonary disease; UD, ulcer disease; DM, diabetes mellitus; LD, liver disease; CCI, Charlson co-morbidity index.

**Fig 1:**
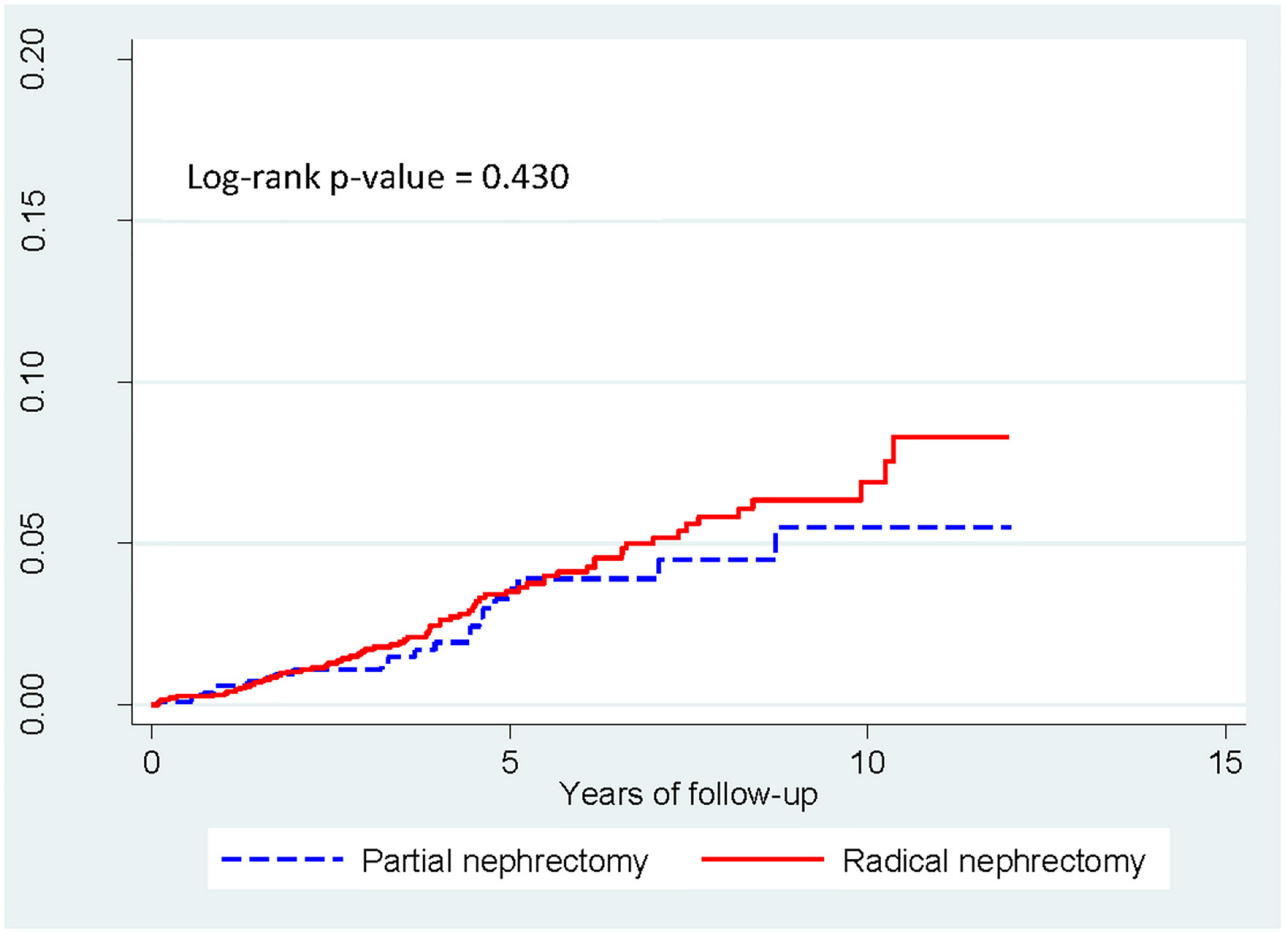
Kaplan-Meier estimates of cumulative risk of having end-stage renal disease (ESRD) by treatment type.

The median follow-up was 48 months for this post-propensity matched cohort (1212 PN and 2424 RN). Seventy patients (2.9%) developed ESRD among patients who underwent RN, for an incidence rate of 6.9 cases per 1000 person-years. In contrast, only 23 patients (1.9%) developed ESRD among patients who underwent PN, for an incidence rate of 5.5 cases per 1000 person-years (Table 2). Despite the higher incidence rate of ESRD among RN, the aIRR (RN/PN) was 1.26 (95% CI 0.78–2.01), which was not statistically significant. The incidence rate increased drastically when the patient’s age was ≥65 years. Nonetheless, the aIRR between RN and PN among older adults was not statistically significant. The cumulative risk of having ESRD by two treatment type is illustrated in Fig 1.

**Table 2:** Incidence rate (cases per 1000 PYs) of (ESRD) among patients with renal cell carcinoma underwent partial or radical nephrectomy, Taiwan, 2000–2011.

	Partial nephrectomy				Radical nephrectomy					
	No	ESRD	PY	IR	No	ESRD	PY	IR	aIRR	95% CI
**Overall**	1210	23	4181	5.5	2420	70	10213	6.9	1.26	(0.78–2.01)
**Age**										
** <45**	229	2	885	2.3	430	5	2042	2.4	1.22	(0.23–6.52)
** 45–64**	588	6	1926	3.1	1199	21	5006	4.2	1.25	(0.50–3.10)
** ≥65**	393	15	1370	11.0	791	44	3165	13.9	1.25	(0.70–2.26)
**Sex**										
** Male**	827	20	2819	7.1	1666	50	6837	7.3	1.06	(0.63–1.78)
** Female**	383	3	1361	2.2	754	20	3376	5.9	2.56	(0.76–8.62)
**CCI**										
** 2**	846	11	3004	3.7	1532	30	7216	4.2	1.16	(0.58–2.32)
** 3**	364	12	1177	10.2	888	40	2997	13.3	1.34	(0.70–2.56)
**DM**	210	9	613	14.7	414	31.0	1567	19.8	1.38	(0.66–2.92)

Abbreviations: ESRD, end-stage renal disease; PYs, person-years; IR, incidence rate; aIRR, adjusted incidence rate ratio; 95% CI, 95% confidence interval; DM, diabetes mellitus.

The results of multivariable Cox proportional hazard regression model (Table 3) revealed that the aHR of developing ESRD among patients who underwent RN compared to those who underwent PN was 1.20 (95% CI 0.75–1.93), which was not statistically significant. Older age and diabetes mellitus were highly associated with the risk of ESRD occurrence.

**Table 3:** Crude (cHR) and adjusted (aHR) hazard ratio of ESRD according to Cox regression model using the post-propensity cohort (n = 3630) in Taiwan, 2000–2011.

Variable	cHR	(95% CI)	*p* value	aHR	(95% CI)	*p* value
**Surgery type (RN: PN)**	1.21	(0.75–1.94)	0.431	1.20	(0.75–1.93)	0.441
**Age**						
** 45–64: <45**	1.67	(0.73–3.85)	0.225	1.36	(0.59–3.15)	0.470
** ≥65: <45**	5.61	(2.56–12.28)	<.0001	3.99	(1.79–8.89)	0.0007
**Sex (Male: Female)**	1.52	(0.95–2.43)	0.082	1.80	(1.12–2.89)	0.016
**DM**	4.37	(2.89–6.59)	<.0001	3.45	(2.25–5.26)	<.0001

Abbreviations: ESRD, end-stage renal disease; DM, diabetes mellitus; RN, radical nephrectomy; PN, partial nephrectomy.

## Discussion

The findings of this Taiwan nationwide population-based study suggest that patients with RCC undergoing RN have a higher incidence rate of developing ESRD compared to patients undergoing PN. However, this association disappears after considering other covariates.

“Do I have less probability of receiving regular hemodialysis by undergoing PN rather than RN?” is a commonly asked question by patients with RCC. However, it is difficult for urologist surgeons to answer this question because the conclusions of population-based studies and clinical trials are inconsistent.

A United States study by Miller et al. using the National Cancer Institute Surveillance Epidemiology and End Results (SEER)-Medicare-linked data identified 10,123 RN and 763 PN patients during 2000 to 2002 and found that patients with PN experienced fewer adverse renal outcomes, including a trend toward less frequent receipt of dialysis services, dialysis access surgery, or renal transplantation [12].

A Canadian study using the Alberta Kidney Disease Network dataset of 1151 post-nephrectomy patients suggests that patients undergoing RN are associated with a hazard ratio (HR) of 1.75 (95% CI 1.02–2.99) for adverse renal outcomes compared to those undergoing PN [13]. Nevertheless, because of the small case number, this Canadian study did not further compare the risk of developing ESRD between the two groups.

Another United States study by Sun et al. using the SEER-Medicare-linked data composed post-propensity matched cohort (840 RN and 840 PN) revealed that patients with RN had 1.9-, 1.3-, 1.8-, and 1.8-fold higher risk of developing chronic kidney disease (CKD), acute renal failure, chronic renal insufficiency and anemia in CKD, and ESRD, respectively. Sun et al. demonstrate a higher risk (HR = 1.76, 95% CI 0.97–3.19) of developing ESRD among patients with RCC aged ≥65 years old who underwent RN. Yet the higher risk is not statistically significant [14]. They further confined the study population to patients treated between 2001 and 2005 and found significantly higher risk of having ESRD among patients who underwent RN (HR = 2.45, 95% CI 1.15–5.23) [14]. One limitation was that the study participants were only those aged ≥65 years old.

The Canadian Ontario study linking the Ontario Cancer Registry data and the Canadian Institute of Health Information Discharge Abstract Data observed 11,937 patients who underwent nephrectomy and found that PN patients (18%) was associated with a decreased likelihood of CKD (HR = 0.48, 95% CI 0.41–0.57) and ESRD (HR = 0.44, 95% CI 0.25–0.75) compared to RN patients [15].

However, the findings of the EORTC randomized trial 30904, the only randomized trial of RN versus PN completed to date, reveal different messages. Of the 259 patients randomized to RN and 255 patients randomized to PN, PN substantially reduced the incidence of at least moderate renal dysfunction (eGFR <60) compared to RN. However, the incidence of advanced kidney disease (eGFR <30) and kidney failure (eGFR <15) was relatively similar in the two treatment arms [18].

The findings of this study are more consistent with the findings of Sun et al. [14], wherein patients with RCC undergoing RN had a higher incidence of developing ESRD than their PN counterparts, which disappeared after controlling for other covariates. However, an aIRR 1.26, even non-significant, indicated in this study should be carefully interpreted. Patients who undergo donor nephrectomy generally have favorable ESRD outcomes with a minor proportion developing ESRD, although studies have shown this is somewhat higher than the general healthy public. Therefore, changing the comparison group for full nephrectomy from two functioning renal units to somewhat less than two would lead us to expect a smaller effect. The variability around the effect measure is also important to consider. A pool of PN patients including those with minor renal functional impairment, solitary kidneys, or other relative or imperative conditions could contribute to non-significance.

With regard to PN, Lane et al. reminded that duration of renal ischemia is the strongest modifiable surgical risk factor for decreased renal function after PN and efforts to limit ischemic time and injury should be pursued in open and laparoscopic PN [19]. A multi-institutional study also suggested PN in a solidary kidney is at risk of post-operative CKD stage V and hemodialysis. Pre-operative altered renal function and post-operative complications are the main predictive factors of permanent CKD stage V [20].

This study is the first Asian study on this issue. The strengths of this study include the inclusion of all age groups and the use of propensity score to adjust the indication bias. Nonetheless, several limitations should be noted in interpreting the findings. First, information on the staging of RCC is not available in the Taiwan NHI claims data. It is possible that some patients have progressed and developed metastases, with the need for a medical treatment, and the risk to increase renal insufficiency using some targeted therapies. Nonetheless, as indicated by Yap et al., the initial stage of RCC does not affect the development of ESRD [15]. If patients with RCC with larger tumor size or advanced stage do have higher risk of developing ESRD, the RN group will include more patients with RCC with larger tumor size or more advanced stage. Thus, the risk of ESRD among RN group will be overestimated and will not change the conclusion.

Second, the information on pre-operative renal function (e.g., serum creatinine level and estimated glomerular filtration rate), differences between imperative and elective indications of conservative surgery, as well as operative parameters that could have influenced the renal history and history of solitary kidney are also not available in the Taiwan NHI claims data. The diagnosis of CKD recorded in the claims data can be used to exclude patients with CKD. As suggested by Lane et al., surgically induced CKD may be associated with a lower risk of progression than medical CKD [21]. In other words, many patients with RCC may have deteriorated renal function but are not diagnosed by physicians and are still included as the study, thereby increasing the risk of developing ESRD. However, the prevalence of medical CKD is unlikely to be differentially distributed in the PN or RN groups and will therefore not bias the conclusion of this study.

Third, the median follow-up is too short (48 months) to conclude to a long-term risk of ESRD, as CKD can appear several years after the surgery. Fourth, some of the risk factors of ESRD, such as cardiovascular events, hypertension or BMI have not been included in the multivariate analysis. Fifth, as no information was available for stage 3 CKD, this study examined only one outcome, i.e., ESRD.

In conclusion, this Taiwan nationwide population-based retrospective cohort study suggests that patients with RCC undergoing RN do not have significantly higher risk of developing ESRD compared to those undergoing PN.
