# Correction: Risk of End-Stage Renal Disease after Cancer Nephrectomy in Taiwan: A Nationwide Population-Based Study

**DOI:** 10.1371/journal.pone.0130647

**Published:** 2015-06-19

**Authors:** Wei-Yu Lin, Fu-Wen Liang, Tsung-Hsueh Lu

The affiliation for the first author is incomplete. Wei-Yu Lin is also affiliated with: Chang Gung University of Science and Technology, Chia-Yi, Taiwan and Chang Gung University, Taoyuan, Taiwan.
